# Encapsulation of Bromelain in Combined Sodium Alginate and Amino Acid Carriers: Experimental Design of Simplex-Centroid Mixtures for Digestibility Evaluation

**DOI:** 10.3390/molecules27196364

**Published:** 2022-09-27

**Authors:** Philipi Cavalcante Ricardo, Ricardo Lima Serudo, Ştefan Ţălu, Carlos Victor Lamarão, Henrique Duarte da Fonseca Filho, Jaqueline de Araújo Bezerra, Edgar Aparecido Sanches, Pedro Henrique Campelo

**Affiliations:** 1 Graduate Program in Materials Science and Engineering (PPGCEM), Federal University of Amazonas (UFAM), Manaus 69067-005, AM, Brazil; 2 Higher School of Technology (EST), State University of Amazonas (UEA), Av. Djalma Batista 2470, Manaus 69050-300, AM, Brazil; 3 The Directorate of Research, Development and Innovation Management (DMCDI), Technical University of Cluj-Napoca, 15 Constantin Daicoviciu St., 400020 Cluj-Napoca, Romania; 4 School of Agrarian Science, Federal University of Amazonas, Manaus 69067-005, AM, Brazil; 5 Laboratory of Synthesis of Nanomaterials and Nanoscopy (LSNN), Department of Physics, Federal University of Amazonas (UFAM), Manaus 69067-005, AM, Brazil; 6 Federal Institute of Education, Science and Technology of Amazonas (IFAM), IFAM Analytical Center, Manaus Centro Campus, Manaus 69067-005, AM, Brazil; 7 Department of Food Technology, Federal University of Viçosa (UFV), Viçosa 36570-900, MG, Brazil

**Keywords:** bromelain, alginate, gastric digestion, encapsulation, controlled release, bioavailability

## Abstract

Bromelain has potential as an analgesic, an anti-inflammatory, and in cancer treatments. Despite its therapeutic effects, this protein undergoes denaturation when administered orally. Microencapsulation processes have shown potential in protein protection and as controlled release systems. Thus, this paper aimed to develop encapsulating systems using sodium alginate as a carrier material and positively charged amino acids as stabilizing agents for the controlled release of bromelain in in vitro tests. The systems were produced from the experimental design of centroid simplex mixtures. Characterizations were performed by FTIR showing that bromelain was encapsulated in all systems. XRD analyses showed that the systems are semi-crystalline solids and through SEM analysis the morphology of the formed systems followed a pattern of rough microparticles. The application of statistical analysis showed that the systems presented behavior that can be evaluated by quadratic and special cubic models, with a *p*-value < 0.05. The interaction between amino acids and bromelain/alginate was evaluated, and free bromelain showed a reduction of 74.0% in protein content and 23.6% in enzymatic activity at the end of gastric digestion. Furthermore, a reduction of 91.6% of protein content and 65.9% of enzymatic activity was observed at the end of intestinal digestion. The Lis system showed better interaction due to the increased stability of bromelain in terms of the amount of proteins (above 63% until the end of the intestinal phase) and the enzymatic activity of 89.3%. Thus, this study proposes the development of pH-controlled release systems aiming at increasing the stability and bioavailability of bromelain in intestinal systems.

## 1. Introduction

Bromelains are proteins found in pineapple (*Ananas comosus*) with beneficial [1] and proteolytic activity widely discussed in scientific literature, presenting therapeutic properties and being traditionally used as an anti-inflammatory and healing agent [2,3,4,5]. Promising effects in burn debridement have been recently reported, such as an analgesic, anti-cancer agent, and more recently, as a nutraceutical to prevent severe COVID-19 [6,7,8]. Recently, bromelain was found to be beneficial for either single or multi-targeted therapy in gastric and skin carcinoma, by inhibiting cancer cell growth. The authors verified that the presence of peroxidase enhanced its biological efficiency [9]. Moreover, bromelain and acetylcysteine presented synergistic activity resulting in dissolution of tumor-produced mucin both in vitro and in vivo [10]. Bromelain was also reported in the hydrolysis of synthetic proenkephalin fragments after basic amino acid residues flanking the enkephalin sequences. In vivo studies showed that oral administration of bromelain reduced jejunum proenkephalin levels and increased the serum enkephalin in mice [11]. The joint potential therapeutic role of bromelain and curcumin was also reported in the prevention of severe COVID-19 [12]. Despite the pharmacological effects [13], the oral administration of bromelain is still a challenge due to its degradation in the stomach, which is associated with the low pH and digestive enzymes [14,15]. Recent advances and insights into bromelain processing, pharmacokinetics, and therapeutic uses have also been reported [16].

Several materials have been evaluated aiming at the stabilization and protection of proteins for pharmaceutical application to prevent degradation by digestive processes. Among the materials used in the formulation of protein systems, sodium alginate is one of the most applied due to its physical and chemical properties [17,18,19]. The impact of pH and thermal treatment were previously evaluated by the encapsulation and release of egg white protein in alginate microgels [20]. On the other hand, the effect of pH on microgel stability, protein retention, and protein release were evaluated based on the protein encapsulation in alginate hydrogel beads [21]. The pH-response double network alginate/kappa-carrageenan hydrogel beads was evaluated under controlled protein release based on the influence of the crosslinking agent [22]. A formulation based on alginate/carrageenan microgels was recently developed to encapsulate, protect, and release immunoglobulins [23]. As bromelain is a polymer of natural origin, and sodium alginate has interesting biological properties such as non-toxicity, low immunogenicity, as well as cellular biocompatibility and biodegradability in the human body. In addition, it is a polysaccharide presenting a negative charge and crosslinking ability [24,25,26]. Sodium alginate can reduce enzyme activity due to possible electrostatic interactions in its network. Therefore, the use of additives represents a viable alternative for the development of proteolytic enzyme formulation [27,28].

Among the additives used in protein stabilization, amino acids have great potential as they are natural components of proteins. In addition, they interact to increase the melting temperature, reducing solution viscosity, suppressing protein aggregation, interacting in protein binding, and presenting a buffering capacity [29,30,31]. Among the essential amino acids, l-lysine, l-arginine [32], and l-histidine are known to be positively charged amino acids. Lysine has been extensively used due to its ability to decrease interfacial tensions and increase electrostatic repulsion [33,34,35,36,37]. Arginine has been used as a solubilizing agent during purification steps and as a highly effective excipient in suppressing protein–protein interaction and protein aggregation [38,39]. Histidine [40] is often used as a buffering agent, in viscosity reduction, and as an antioxidant in various formulations [29,36]. Thus, the present work investigated the use of positively charged amino acids combined with sodium alginate in the formulation and stabilization of bromelain in in vitro digestion systems. Furthermore, the possible correlation between the amino acids and the maintenance of the proteolytic activity was evaluated after the digestion assays using an experimental design of mixtures.

## 2. Materials and Methods

### 2.1. Materials

Bromelain was purchased from Hi-Media (Mumbai, India). Sodium alginate was purchased from Êxodo Química (São Paulo, Brazil). L-lysine, l-arginine, and l-histidine were purchased from LabSynth (São Paulo, Brazil). Azo-casein and Coomassie Brilliant Blue R-250 were acquired from Sigma-Aldrich (São Paulo, Brazil). All other reagents were purchased with an analytical grade.

### 2.2. Microcapsules Synthesis

Systems were synthesized using a mixture (1:1:0.5; sodium alginate/amino acids (pure l-lysine, l-arginine, and l-histidine and their mixtures)/bromelain) for each 100 mL of solution. A mass of 5.0 g of sodium alginate was hydrated in 250 mL of deionized water overnight under magnetic stirring. Then, 5.0 g of amino acids was added to 250 mL of deionized water until complete dissolution. A portion of 2.5 g of bromelain was added to the solution containing amino acids and stirred until complete solubilization. Finally, the synthesis solution was prepared by mixing the hydrated alginate solution and the amino acids solution containing bromelain, resulting in the ratio described before. A volume of 500 mL of solution was used for the microcapsules formation on a spray dryer (LabMaq model MSDi 1.0, Ribeirão Preto, São Paulo, Brazil) with a dual fluid atomizing nozzle, blowing flow rate of 0.75 m^3^.min^−1^, inlet temperature of 120 °C, solution injection rate of 0.4 L.h^−1^, spray air injection flow rate of 35 L.min^−1^, and 1.2 mm atomizer. Synthesized microparticles were stored in sealed glass vials and stored at −20 °C.

### 2.3. Design of Experiments and Statistical Analysis

Amino acid combinations were prepared according to the simplex-centroid mixture design with 2^q^-1 combinations, where *q* is the number of tested components (amino acids), with a sum equal to 1 and two repetitions of the central point (7C) for error calculation. The experimental domain consisted of components X1 (l-lysine), X2 (l-arginine), and X3 (l-histidine) between zero and one (0 ≤ Xi ≤ 1; ∑Xi = 1) randomly produced, as shown in Table 1.

Systems without bromelain were synthesized to correct the background signal of the produced systems. Mixture regression analysis was performed on the digestibility data to determine the estimated coefficients and the significance of the model terms, the F test, and the coefficient of determinations (R^2^).

Results were initially fitted to all available mixture regression models of increasing complexity, from linear to special cubic. The significance of the model, the significance of lack of fitting, as well as the adjusted R^2^ value were used to evaluate the model’s fitting. Adjusted R^2^ describes the proportion of variation in responses explained by the model. The value was adjusted to the number of terms in the model. The models were then reduced (if any), considering only significant terms. Finally, analysis was considered more adequate (due to the insignificant lack of fitting and the higher adjusted R^2^) as the quadratic model or special cubic model, according to Equations (1) (quadratic model) and (2) (special cubic model).

Y = β_1_X_1_ + β_2_X_2_ + β_3_X_3_ + β_12_X_1_X_2_ + β_13_X_1_X_3_ +β_23_X_2_X_3_ (1)

Y = β_1_X_1_ + β_2_X_2_ + β_3_X_3_ + β_12_X_1_X_2_ + β_13_X_1_X_3_ +β_23_X_2_X_3_+ β_123_X_1_X_2_X_3_ (2)

where Y is the predicted analysis response; β_1_, β_2_, and β_3_ are the estimated coefficients of each linear effect term; β_12_, β_13_, and β_23_ are the binary interaction effect terms, and β_123_ is the ternary interaction effect term. Contour and surface plots were generated after obtaining the estimated model equation. Graphics showed a response related to three components based on the model equation. Two new ternary mixtures were used to prepare two new respective emulsions (*n* = 2). Finally, the model was validated by comparing the mean responses of these emulsions with the respective values predicted by the model equations. Experimental design, data analysis, and contour and surface plots were developed using the software Statistica^®^ (version 10.0, Statsoft Inc., Tulsa, OK, USA). The applied confidence level was α = 0.05.

### 2.4. Moisture Content Analysis

Moisture content measurements were performed on a Marte Model ID50 (São Paulo, SP, Brazil) moisture measurement scale with an infrared heat source through gravimetric measurement of drying at 105 °C. Capsules were dried until they reached constant mass.

### 2.5. Solid Recovery Analysis

Mass yield (%) was obtained after the spray drying process based on Equation (3) (mass yield (%) = (total mass after drying/total solid content in feed)), in which the total solid content corresponds to the mass amount of alginate, amino acids, and bromelain used in the production of the system solutions, and the total mass after drying corresponds to the amount collected in the spray dryer receiver bottle.

### 2.6. FTIR Analysis

Possible interactions between alginate, bromelain, and amino acids were evaluated on a Fourier-transform Infrared Spectrophotometer (IR Prestige-21, Shimadzu, Kyoto, Japan) with IRsolution software version 1.6 at the ILUM Laboratory-HUB (UEA). Translucent pellets were prepared by mixing 140 mg of infrared grade KBr and 10 mg of sample. Scans were applied in the range 400–4000 cm^−1^, transmittance scanning mode, Happ-Genzel apodization, 64 scans, and resolution of 1.0 cm^−1^.

### 2.7. XRD Analysis

XRD measurements were performed at the IFAM/CMC Analytical Center on an X-ray diffractometer (Shimadzu model XRD-7000, Kyoto, Japan), K_α_Cu, 50 kV, and 100 mA. Measurements were obtained in the stepscanning mode, with an angular increment of 0.02° and 5 s/step, in the range from 2θ = 20°–100°.

### 2.8. SEM Analysis

The morphology of the developed capsules was evaluated at the Laboratory of Electron Microscopy and Analysis (LMEA/INPA) using a Carl Zeiss microscope model SIGMA (Jena, Germany) equipped with a field electron gun (SEM-FEG). Samples were coated with a thin gold film and images were taken at 25 °C using 2 kV.

### 2.9. Protein Content Analysis

Protein quantification was performed by adding 500 µL of Coomassie Brilliant Blue G-250 solution to 500 µL of sample in a 2 mL microtube, with subsequent vortexing for 30 s and centrifugation at 10,000 rpm for 10 min at 4 °C. Then, photometric analysis was performed at 595 nm in a UV-VIS spectrophotometer (Shimadzu, Kyoto, Japan). Results were expressed as a free percentage in relation to the initial amount of bromelain.

### 2.10. Enzyme Activity Analysis

The enzymatic activity was performed by the method of quantification of azo-casein digestion. A volume of 125 µL of bromelain solution or sample was mixed with 125 µL of 1.0% (*w/v*) azo-casein solution in a 2.0 mL Eppendorf tube, vortexed for 30 s, and taken to thermoblock for 60 min at 37 °C for the proteolytic reaction. After 60 min, 750 µL of 5% trichloroacetic acid (m·V^−1^) was added to stop the reaction and coagulation of unbroken proteins. The solution was separated by centrifugation at 10,000 rpm for 10 min at 4 °C. Then, 500 µL of the supernatant was added to 500 µL of 0.5 mol·L^−1^ NaOH solution and taken for photometric analysis at 440 nm on a UV-VIS spectrophotometer (Shimadzu, Kyoto, Japan). The enzyme activity was determined by correlation with a standard curve, measured by the amount of chromophores resulting from the digestion of azo-casein at different concentrations in 60 min at 37 °C [41].

### 2.11. SFG and SFI Digestibility Analyzes

The in vitro simulated digestibility study (gastric and intestinal phases) was based on the method described elsewhere [42], without the presence of digestive enzymes and bile salts, since bromelain has proteolytic activity, and the activity of the systems can be masked by digestive enzymes.

For digestion in gastric fluid simulation solution (SFG) and intestinal fluid simulation solution (SFI), 125 mL Erlenmeyer flasks were used in which 100 mg of the corresponding system was added to 10 mL of gastric fluid solution and placed in a shaker incubator at 150 rpm and 37 °C for 2 h. Then, the Erlenmeyer corresponding to SFG was removed from the shaker and an aliquot was collected for protein dosage and enzymatic activity corresponding to the gastric phase. A SGF 1.25X was prepared as follows: 6.9 mM KCl, 0.9 mM KH_2_PO_4_, 25 mM NaHCO_3_, 47.2 mM NaCl, 0.1 mMMgCl_2_, and 0.5 mM (NH_4_)_2_CO_3_. Then, a volume of 0.3 M CaCl_2_ was added to obtain a final concentration of 0.075 mM CaCl_2_. Finally, the SFG was adjusted to pH = 3.0 [42,43].

After digestion in gastric fluid, the pH of the remaining solution was adjusted to 7.0 using 0.1 M NaOH. Then, 10 mL of SFI solution was added to the Erlenmeyer flasks and maintained under the same stirring conditions, temperature, and time used in gastric digestion. A SFI 1.25X was prepared as follows: 6.8 mM KCl, 0.8 mM KH_2_PO_4_, 85 mM NaHCO_3_, 38.4 mM NaCl, and 0.33 mMMgCl_2_. Then, a volume of 0.3 M CaCl_2_ was added to obtain a final concentration of 10 mM CaCl_2_. After 2 h under SFI, an aliquot was collected and used for protein dosage and quantification of enzymatic activity corresponding to gastric digestion followed by intestinal digestion [42,43].

## 3. Results and Discussion

### 3.1. Regression Models

The responses obtained for the design of mixtures are shown in Table 2. Results were used in the production of linear, quadratic, special cubic, and fully cubic regression models where the analysis of residue graphs and error were generated to verify the quality of the models. The best model has low standard deviation, low predicted sum squared, and high predicted R^2^. Thus, Table 3 presents the regression coefficients for each response while Table 4 presents the analysis of variance of model fitting, where good fitting for the special cubic model is observed in most responses except protein content, which showed good fitting for quadratic regression models.

### 3.2. Effects of Amino Acid Combination on Protein Content

Seven different systems containing encapsulated bromelain within sodium alginate/amino acid particles, as well as their mixtures, were produced. In addition, two repetitions of the central point systems for error calculations were also performed. Unloaded bromelain was used as a control sample, showing 26.0% of protein content after 120 min under gastric digestion conditions and 8.4% after gastric digestion followed by intestinal digestion.

Table 2 shows the behavior of the systems in relation to the unloaded bromelain. The order of protein content after gastric digestion was Arg > LAH > LH = AH > LA > His > Lis. On the other hand, after gastric digestion followed by intestinal digestion the order was Lis > AH > LH > His > LAH > LA > Arg. The systems formed by the combination of alginate and amino acids presented protein content ranging from 63.6% to 90.1% after gastric digestion, and from 31.7% to 59.7% after gastric digestion followed by intestinal digestion. These values were significantly higher than those of unloaded bromelain.

Table 3 presents the analysis of variance for the applied models, showing that the quadratic models well described (R^2^ > 0.94) the interactions between the combination of amino acids and the impact on the digestibility of the systems.

Table 4 presents the values of the regression coefficients (β_1_, β_2_, and β_3_) and adjusted R^2^ for the produced models. The coefficients with statistical significance were presented (*p*-value < 0.05). Our results showed that the Arg system considerably influenced the protein content after digestion in gastric fluid, reaching 90.1%. Similar behavior was not observed in the combination of arginine with lysine and histidine, or the mixture between amino acids (β_12_, β_23_, and β_123_), which did not present statistical significance (*p*-value > 0.05).

**Figure 1:**
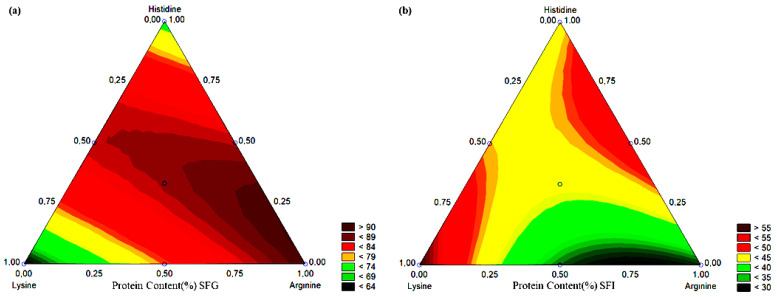
Contour curve of the tested systems for protein content (%) in (**a**) SFG and (**b**) SFI.

Figure 1a presents the level curve for the variation of the protein content in the synthesized systems, where the maximum release was observed in the extremes composed of arginine and combinations. The Lis and His systems showed 63.6% and 72.3% of protein content, respectively, after digestion in gastric fluid. These values were lower than that of 90.1% found in the Arg system, but considerably higher than that of unloaded bromelain (26.0%).

Amino acids may have both antioxidant and anti-aggregating properties. Arginine tends to interact with proteins in order to avoid aggregation, which can directly influence the protein solubility and consequent diffusion through alginate networks [38,44,45]. This behavior was not observed in the other amino acids and their mixtures, which tended to interact in different ways with proteins and alginate. As a result, lower diffusion rates and protein solubility were observed when compared to the systems containing arginine [46,47,48].

The Lis system presented greater influence on the maintenance of protein content after digestion in gastric fluid followed by intestinal fluid, reaching 59.7%. This behavior was not observed in digestion in gastric fluid, where the combination of two amino acids was statistically relevant (*p*-value < 0.05). However, it presented a negative tendency when used by mixing lysine with another amino acid (β_12_ and β_13_). Similar behavior was not observed for the combination of three amino acids. Figure 1b shows the level curve for the variation of the protein content in the synthesized systems where maximum retention was observed in the extremes composed of lysine and histidine and combinations. The LA, LH, and AH systems showed, respectively, 33.0%, 46.2%, and 51.5% of protein content after digestion in gastric fluid followed by intestinal fluid. Values lower than 59.7% were achieved in the Arg system, which is significantly higher than that of unloaded bromelain (8.4%).

The digestion in gastric fluid followed by intestinal fluid showed the influence of arginine on the destabilization of bromelain under pH variation. However, the protein content achieved by the Arg system after intestinal digestion was 31.7%, which was considerably higher than that of unloaded bromelain. This protection factor can be attributed to the use of alginate as a carrier, which reduced the influence of the gastric solution on bromelain since sodium alginate presents an antiacid property in low pH systems [27,28]. Considering l-lysine and l-histidine, protein aggregation was less pronounced, resulting in less protein stress due to pH variation.

The use of amino acids with alginate matrices is not common. However, the known properties they present as a buffering agent, protein anti-aggregating agent, and antioxidant are especially useful in spray dryer syntheses [29], as well as in systems with high pH variation such as the gastric system. Due to the positive polarization that they present in acidic media, they are expected to contribute synergistically with sodium alginate (positively charged) in a greater ionic repulsion in the gastric environment and consequent reduction of diffusive effects by electrostatic potential. However, despite the synergistic contributions expected from the use of positive amino acids, l-arginine presented itself as a lysis protein catalyst, most likely due to its anti-aggregation capacity with consequent exposure of the protein.

Thus, the use of negatively charged or even uncharged amino acids can bring benefits or even harm in combination with sodium alginate (whether electrically synergistic or not), due to the properties of amino acid–protein interaction. However, more studies should be carried out to evaluate the effectiveness of the combination of charged or uncharged amino acids.

### 3.3. Effects of Amino Acid Combination on Residual Enzyme Activity

The tested systems presented enzymatic activity of unloaded bromelain at pH 7.0 around 10.5 U.mL^−1^, which was considered as 100% for the evaluated systems. Thus, the residual enzyme activity (%) of the unloaded bromelain after 120 min under gastric digestion conditions and gastric digestion followed by intestinal digestion were, respectively, 76.4% and 34.1%.

Table 2 shows the behavior of the systems in relation to the unloaded bromelain. The order of residual enzymatic activity (%) after gastric digestion was LH > Arg > LA > Lis > His > AH > LAH. On the other hand, after gastric digestion followed by intestinal digestion the order was found as Lis > LH > His > AH > LA > Arg > LAH. The systems formed by the combination of alginate and amino acids presented residual enzymatic activity (%) ranging from 13.9% to 68.5% after gastric digestion, which presented lower values when compared to the unloaded bromelain. These values were found from 3.5% to 89.3% after gastric digestion followed by intestinal digestion, presenting a minimum value lower than that of the unloaded bromelain, and a maximum value considerably higher.

Table 4 shows that the special cubic models were able to describe (R^2^ > 0.96) the interactions between the amino acid combination and the impact on the retention of bromelain proteolytic activity.

The combination of l-lysine and l-histidine (LH) presented a positive influence on the retention of protein activity after digestion in gastric fluid, presenting the highest retention. Similar behaviors were observed in systems without amino acid combination: the Arg system presented the greatest influence.

**Figure 2:**
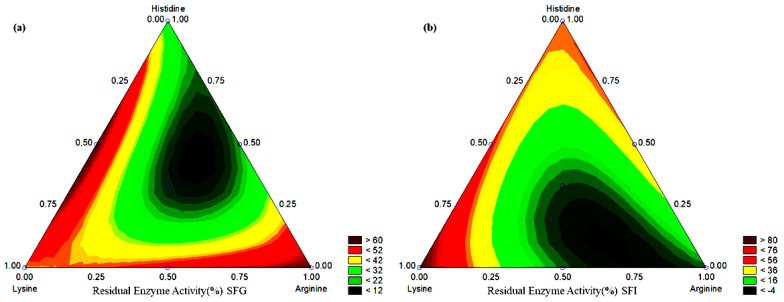
Contour curve of the tested systems for residual enzyme activity (%) in (**a**) SFG and (**b**) SFI.

Figure 2a shows the level curve for the variation of residual enzymatic activity (%) in the analyzed systems, where maximum activity was observed in the extremes composed of pure amino acids and minimum region in the systems combining amino acids. The minimum region was observed at the central point. With the exception of the LH system, the combination effects between amino acids resulted in negative effects on residual enzyme activity (%), showing the higher coefficient values in Table 4.

Amino acids may have different properties of protein protection and stabilization. In particular, the anti-aggregation and solubilization function of l-arginine can result in considerable exposure of the active sites of bromelain, enhancing the enzymatic activity in the gastric phase. Considering the combination of amino acids, l-lysine and l-histidine seem to have synergistic effects in the maintenance of residual enzyme activity (%), while the mixture between the other amino acids has negative effects.

Our results showed that lysine presented greater influence on the maintenance of residual protein activity (%) after digestion in gastric fluid followed by intestinal fluid, reaching 89.3% (Lis system). The combination behavior between amino acids showed a negative tendency, except for the combination of arginine and histidine. Figure 2b shows the level curve for the variation in the maintenance of enzymatic activity (%) in systems where maximum values were observed in the extremes composed of lysine and histidine, and combinations and minimum values of combination of lysine and pure arginine. The LAH, Arg, and LA systems showed 3.5%, 5.7%, and 6.3%, respectively, of residual enzyme activity (%) after digestion in gastric fluid followed by intestinal fluid. These values were lower than that of unloaded bromelain (8.4%). However, the systems His, LH, and Lis presented 50.7%, 55.4%, and 89.3% of maintenance of enzymatic activity (%).

**Figure 3:**
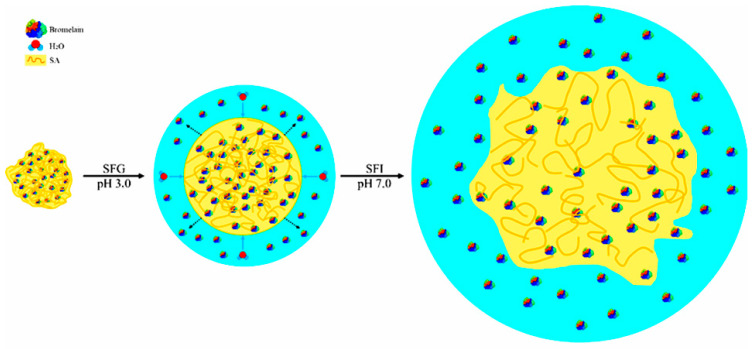
Dry system (**left**); process of rehydration and protein diffusion in contact with SFG in pH 3.0 (**center**) and processes of degradation of the systems with release of non-diffuse proteins from the gastric phase in pH 7.0 (**right**).

Arginine plays an important role in the destabilization of bromelain under pH variation. The presence of sodium alginate was not enough to reduce the stress to pH variation. In this case, arginine seems to strongly unbalance bromelain since the protein breakdown process allows a greater surface attack by pH variation [38]. Figure 3 shows a protein stress scheme by protein breakdown and pH variation.

As observed for the protein content (%), l-lysine and l-histidine presented good protein stabilization properties due to their buffering or antioxidant effect combined with the antacid effects of alginate networks [47,49]. Another possible influencing factor is the greater exposure of proteins due to the disruption of alginate networks at higher pHs, which can increase the protein mobility by exposing their active reaction sites [50,51].

### 3.4. Solid Recovery Analysis

Table 2 shows the behavior of systems where LH > AH > LAH > His > LA > Arg > Lis, ranging from 26.8% (for the Lis system) to 44.6% (for the LH system). These values were lower than those found in other studies and represent a result of adhesion processes to the spray dryer wall and smaller particle sizes [52,53,54].

**Figure 4:**
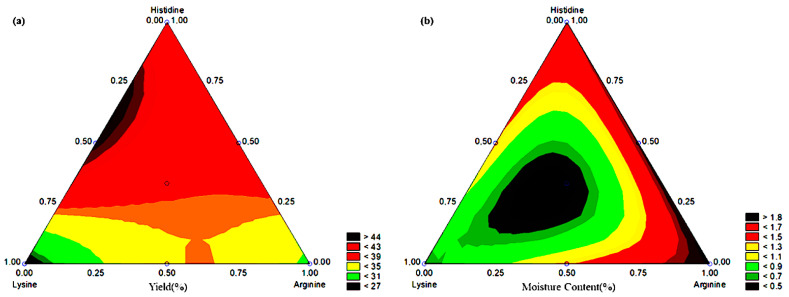
Contour curve of the tested systems in (**a**) yield (%) and (**b**) moisture content (%).

Table 3 shows that the special cubic model fitted the description of yield (%) with *p*-value = 0.0251. Table 4 shows that only the combination of l-arginine and histidine (β_23_) was not statistically significant. Furthermore, the combination of the three amino acids negatively impacts yield (%). Figure 4a shows the yield (%) optimization region in the combination of lysine and histidine. In addition, pure histidine tended to increase yield (%).

The combination effects can be associated with a greater stereo complexation of the alginate network to the amino acids. A diffusion can occur through the alginate networks, contributing to lower aerosolization of the particles and resulting in the adhesion of powder to the spray dryer wall [55].

### 3.5. Moisture Content Analysis

Table 2 shows the behavior of systems where Arg > AH > His > LA > LH > Lis > LAH, ranging from 0.5% (for the LAH system) to 1.92% (for the Arg system). These values were relatively lower than those found in other studies [52,55].

Table 3 shows that the special cubic model fitted the description of the moisture content (%). However, the *p*-value was found in the limit of statistical significance (0.0498). Table 4 shows that only the combination of l-arginine (β_2_), histidine (β_3_), and the combination of the three amino acids (β_123_) was statistically significant, where the combination of amino acids negatively impacted the amount of moisture in the systems. Figure 4b shows the moisture reduction optimization region in the combination of the three amino acids (at the central point).

Some studies reported the use of amino acids in the reduction of aggregation processes between particles and recrystallization induced by the increase of humidity. The addition of the amino acids isoleucine, valine, and methionine generated storage-stable systems with 60% and 70% humidity for inhalation drugs [56]. A similar effect was reported in the use of l-leucine and l-isoleucine for stabilization of trehalose carrier in systems with 50% humidity [57]. Therefore, the use of amino acids in the formation of microencapsulated systems has a great advantage against moisture-induced recrystallization processes.

### 3.6. FTIR Analysis

**Figure 5:**
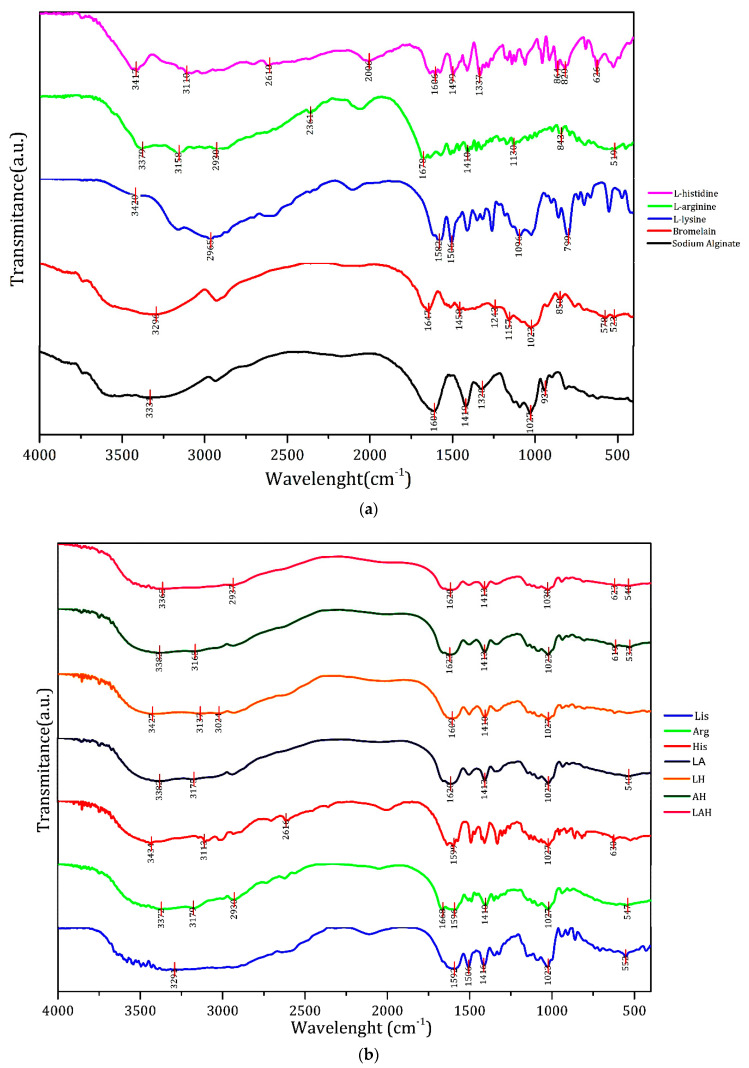
(**a**) FTIR spectra of the individual compounds of the developed systems and (**b**) FTIR spectra of the developed systems.

Figure 5 shows the spectra of the individual compounds of the developed systems. L-lysine was characterized by the peaks at 3420 cm^−1^ (OH stretching), 2965 cm^−1^ (CH asymmetric stretching), 1582 cm^−1^ (NH_3_^+^ asymmetric scissoring), 1506 cm^−1^ (NH_3_^+^ symmetric scissoring), 1096 cm^−1^ (CH stretching), and 790 cm^−1^ (COO^−^ scissor) [58].

The l-arginine spectrum presented peaks at 3379 cm^−1^ (NH stretching), 3158 cm^−1^ (NH_2_ stretching), 2930 cm^−1^ (CH stretching), 2361 cm^−1^ (CH and COO^−^ stretching), 1678 cm^−1^ (COO^−^ asymmetric stretching), 1410 cm^−1^ (COO^−^ symmetric stretching), 1139 cm^−1^ (NH_2_ elongation), 843 cm^−1^ (CH stretching), and 510 cm^−1^ (NH elongation) [59,60].

The l-histidine spectrum presented peaks at 3417 cm^−1^ (OH elongation), 3110 cm^−1^ (CH elongation), 2610 cm^−1^ (N–H, NH_2_, and NH_3_^+^ elongation), 2006 cm^−1^ (NH elongation), 1606 cm^−1^ (COO asymmetric elongation and NH_3_^+^ scissoring), 1499 cm^−1^ (CH_2_ scissoring), 1337 cm^−1^ (ring stretching), 864, 829, and 626 cm^−1^ (CH out-of-plane stretching) [61,62].

The FTIR peaks of bromelain were found at 3296 cm^−1^ (OH elongation), 1647 cm^−1^ (CO elongation), 1458, 1243, and 1157 cm^−1^ (CN elongation), 1023 cm^−1^ (CO elongation), 850 cm^−1^ (CH out-of-plane strain), and 578 cm^−1^ and 523 cm^−1^ (ring out-of-plane strain) [63,64,65]. The sodium alginate peaks were found at 3331 cm^−1^ (OH elongation), 1609 cm^−1^ (COO elongation), 1410 cm^−1^ (COO elongation), 1320 cm^−1^ (CO stretching), 1027 cm^−1^ (CH stretching), and 937 cm^−1^ (CH stretching) [66].

The systems containing only pure amino acids showed more defined characteristic peaks, such as those at 3291, 1416, and 1023 cm^−1^, which were assigned to the sodium alginate phase. The peaks at 1592, 1506, and 552 cm^−1^ (NH stretching) corresponded to the l-lysine of the system Lis. The peaks at 3372, 3170, and 547 cm^−1^ (NH stretching) were assigned to the l-arginine, and the peaks at 2930 cm^−1^, 1668 cm^−1^ (COO asymmetric stretching), 1596, 1410, and 1027 cm^−1^ corresponded to the sodium alginate phase in the Arg spectrum. The peaks at 2616 cm^−1^ (NH stretching) and 639 cm^−1^ (NH stretching) corresponded to the l-histidine, and the peaks at 3434 cm^−1^ (OH elongation), 3113 cm^−1^ (CH elongation), 1599 cm^−1^ (COO elongation), and 1027 cm^−1^ were assigned to the sodium alginate phase in the system His.

The mixtures between one or more amino acids reduced the definition of the resulting peaks at 3328, 3175, 1620, 1413, 1027, and 540 cm^−1^ in the system LA; at 3427, 3137, 3024, 1609, 1410, and 1027 cm^−1^ in the system LH; at 3382, 3165, 1623, 1413, 1023, 619, and 533 cm^−1^ in the system AH, and at 3365, 2937, 1620, 1413, 1030, 623, and 540 cm^−1^ in the system LAH.

During the spray drying process, the amino acids tended to diffuse between the droplet formation networks, especially to the smaller ones [67]. Therefore, as observed in the Lis, Arg, and His systems, the amino acids tended to move to regions closer to the particle surface, resulting in greater protection of bromelain. This result was observed because the amino acid acts as a surface barrier and concentrates the protein within the particles [68]. Better-defined vibrational effects were not observed in the two additional amino acid combination systems probably due to the combination of individual amino acid peaks. Specific peaks related to bromelain were not observed, showing its successful encapsulation.

### 3.7. XRD Analysis

**Figure 6:**
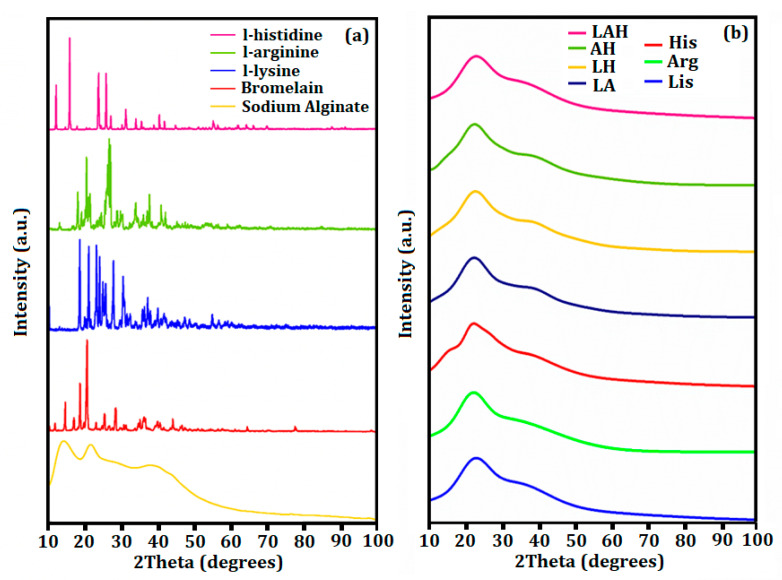
(**a**) XRD patterns of the individual compounds of the developed systems; (**b**) XRD patterns of the developed systems.

Figure 6a presents the XRD pattern of the individual compounds of the developed systems. Amino acids and bromelain presented crystalline behavior with well-defined peaks between 2θ = 10°–50°. Sodium alginate, on the other hand, presented a semi-crystalline XRD pattern with peaks at 2θ = 14.5° (G block) and 2θ = 21.6° (M block), which corresponded to the intermolecular hydrogen bonds in the polyguloromic unit (G block) and in the polymanuromico (M block) plane, in addition to an amorphous halo centered in 2θ = 35.9° [69,70].

Figure 6b presents the XRD patterns of the developed systems. The peak at 2θ = 22.9° corresponded to the M block of sodium alginate, and the amorphous region was found centered at 2θ = 35.6° (which is also characteristic of the sodium alginate phase). For this reason, these results suggested that bromelain and amino acids interacted with the G blocks of the sodium alginate phase through electrostatic interactions with the functional groups COO^−^ resulting in homogeneous and amorphous systems [71].

Developed systems for controlled release of bromelain in the intestines was reported by applying Eudragit L100 [65]. The authors found amorphous characteristics confirmed by XRD analysis, showing the importance of the amorphous property for lower water absorption capacity. The use of carbohydrates in protein microencapsulation has been extensively reported, as well as the use of amino acids to increase the stabilization of tertiary and quaternary structures of proteins [21,29,31,57,72]. Thus, our results indicated the potential of combining these materials to preserve the physical and chemical properties of proteins, especially bromelain.

### 3.8. Morphological Analysis

**Figure 7:**
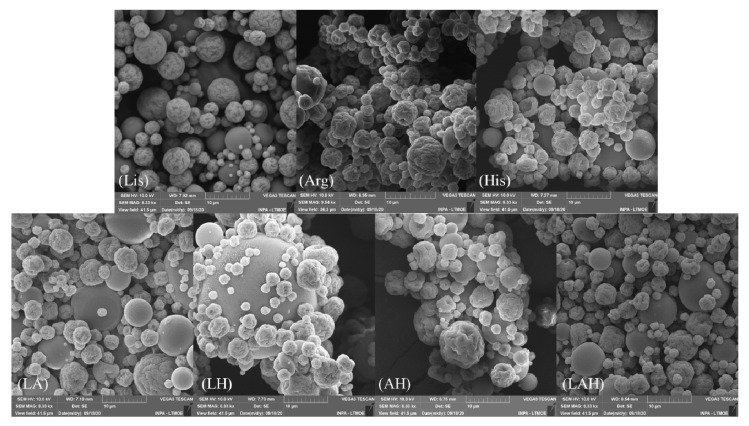
SEM images of the developed systems.

Figure 7 presents the SEM images of the developed systems. Two different morphologies were observed, indicating complex drying processes: particles presented smooth or rough shapes.

The encapsulation process using amino acids can result in different particle morphology [52,56,73]. The roughness shapes can influence the increase of the diffusion rate of the encapsulated systems due to the increase of surface area when compared to uniformly spherical particles [74,75,76].

Previous studies have shown that spray drying processes are influenced by temperature, viscosity, concentration, and spray nozzle size. As a result, different types of morphology have been obtained. In addition, materials used in microencapsulation can generate rounded, rough structures or structures with holes and halos that indicate possible explosion or erosion of particles during drying [76,77,78]. In the formed systems, the particles presented two types of morphology, which can be explained by the diameter of the atomizer used, since a 1.2 mm disperser was used, which induces the production of droplets of greater volume that tend to form rougher structures.

Some studies show that encapsulation using amino acids can also influence the formation of different morphologies. Herein, the spherical capsules had smooth and rough surfaces or had small undulations [52,56,73].

A previous study reported the use of sodium alginate in spray drying insulin encapsulation. The authors obtained two morphologies, where the capsules presented smooth surfaces or with small undulations. The ripples can act to increase the diffusion rate of the encapsulate, by increasing the surface area, when compared to uniformly spherical particles. However, in other cases, extensive divisions induce flattening of the particles, which could reduce the surface area and decrease encapsulate release rates [74,79,80].

## 4. Conclusions

The present study represents the first analysis of the combination of amino acids with sodium alginate to increase the physical and chemical stability of proteins against digestive processes. Our results were based on positively charged amino acids, whose properties aided in a greater delivery of protein and enzymatic activity after gastric and intestinal digestion under in vitro assays. The combined use of these substances directly influenced the enhanced amount of proteins after digestion in SFG and SFI when compared to unloaded bromelain. The amino acids l-lysine and l-histidine presented the greatest capacity for protein stabilization and retention of enzymatic activity for bromelain, showing high potential in the use of pH-controlled release systems to increase the bioavailability of proteins in the intestinal phase.

## Figures and Tables

**Table 1:** A simplex-centroid design with ratios of l-lysine (X1), l-arginine (X2), and l-histidine (X3).

Code	Component Ratio (% m·V^−1^)					Execution Order
	Sodium Alginate	Bromelain	X1	X2	X3	
Lis	1.0	0.5	1	0	0	7(C)
Arg	1.0	0.5	0	1	0	5
His	1.0	0.5	0	0	1	3
LA	1.0	0.5	1/2	1/2	0	7(C)
LH	1.0	0.5	1/2	0	1/2	2
AH	1.0	0.5	0	1/2	1/2	4
LAH	1.0	0.5	1/3	1/3	1/3	1
LAH	1.0	0.5	1/3	1/3	1/3	7(C)
LAH	1.0	0.5	1/3	1/3	1/3	6

The first three systems were labeled using the initial letters corresponding to the amino acid content, i.e., l-lysine = Lis, l-arginine = Arg, and l-histidine = His. In the other systems, the first letter of the corresponding amino acid was used to label the systems: LA = mixture of l-lysine and l-arginine, LH = mixture of l-lysine and l-histidine, AH = mixture of l-arginine and l-histidine, and LAH = mixture of l-lysine, l-arginine, and l = histidine.

**Table 2:** Behavior of the developed systems in relation to the unloaded bromelain.

Code	Yield (%)	Moisture Content (%)	Protein Content (%) SFG	Protein Content (%) SFI	Residual Enzyme Activity (%) SFG	Residual Enzyme Activity (%) SFI
Lis	26.8	0.62	63.6	59.7	43.3	89.3
Arg	33.1	1.92	90.1	31.7	67.5	5.7
His	38.0	1.67	72.3	44.6	27.4	50.7
LA	36.7	1.27	78.3	33.0	50.2	6.3
LH	44.6	1.25	85.8	46.2	68.5	55.4
AH	40.1	1.84	85.8	51.5	19.9	45.9
LAH	38.5	0.61	87.1	41.8	15.1	3.5
LAH	39.7	0.49	87.3	42.4	13.9	3.3
LAH	37.9	0.29	89.8	44.0	13.6	4.8

**Table 3:** Analysis of variance for the applied models.

Source	Degree of Freedom	Adjusted Sum of Square	Adjusted Mean Square	F Value	*p*-Value
*Fitted with special cubic*					
Yield (%)					
Model	6	195.99	32.66539	39.2081	0.0251
Lack-of-fit	0	0.0000	0.0000		
Pure Error	2	1.6663	0.83313		
					
Moisture Content (%)					
Model	6	3.042333	0.507056	19.4026	0.0498
Lack-of-fit	0	0.0000	0.0000		
Pure Error	2	0.0523	0.0261		
					
Residual Enzyme Activity (%) SFG					
Model	6	4058.963	676.4938	1070.36	0.0009
Lack-of-fit	0	0.0000	0.0000		
Pure Error	2	1.264	0.632		
					
Residual Enzyme Activity (%) SFI					
Model	6	8032.828	1338.805	2216.15	0.0005
Lack-of-fit	0	0.0000	0.0000		
Pure Error	2	1.208	0.604		
					
*Fitted with quadratic model*					
PC (%) SFG					
Model	5	648.0382	129.6076	42.4920	0.0055
Lack-of-fit	1	4.5162	4.5162	1.9491	0.2975
Pure Error	2	4.6343	2.3171		
					
PC (%) SFI					
Model	5	585.9594	117.1919	130.7413	0.0010
Lack-of-fit	1	0.0622	0.0622	0.0473	0.8479
Pure Error	2	2.6269	1.3135		

**Table 4:** Regression coefficients (β1, β2, and β3) and adjusted R2 of the produced models.

Response	β_1_	β_2_	β_3_	β_12_	β_13_	β_23_	β_123_	R^2^
Yield (%)	26.8	33.1	38.0	27.2	48.8	*	–118.9	0.9663
Moisture Content (%)	*	1.9	1.7	*	*	*	–27.2	0.9324
Residual Enzymatic Activity (%) SFG	43.3	67.5	27.4	–20.8	132.5	−110.3	–864.4	0.9629
Residual Enzymatic Activity (%) SFI	89.3	5.7	50.7	–164.7	–58.3	70.6	–749.7	0.9878
Protein Content (%) SFG	63.3	89.8	72.1	*	76.4	*	-	0.9994
Protein Content (%) SFI	59.8	31.7	44.6	–51.2	–24.2	52.8	-	0.9475

*: No significant regression coefficient.

## Data Availability

Not applicable.

## Sample Availability

The data used to support the findings of this study are available from the corresponding author upon request.
